# Ethanolic Extract of *Stachys byzantina* Leaf: Optimization of Ultrasonic Probe-Assisted Extraction and Characterization

**DOI:** 10.3390/plants14233636

**Published:** 2025-11-28

**Authors:** Sthefany Lorena Gemaque Dias, Djéssica Tatiane Raspe, Oscar de Oliveira Santos Júnior, Maria Luisa Gonçalves Agneis, Fabio Rodrigues Ferreira Seiva, Vitor Augusto dos Santos Garcia, Lúcio Cardozo-Filho, Camila da Silva

**Affiliations:** 1 Programa de Pós-Graduação em Engenharia Química, Departamento de Engenharia Química, Universidade Estadual de Maringá, Av. Colombo 5790, Maringá 87020-900, PR, Brazil; sthefany.dias@gmail.com (S.L.G.D.); djessicaraspe@hotmail.com (D.T.R.); lcfilho@uem.br (L.C.-F.); 2 Departamento de Química (DQI), Universidade Estadual de Maringá (UEM), Av. Colombo, Maringá 87020-900, PR, Brazil; oosjunior@uem.br; 3 Departamento de Química e Bioquímica, Instituto de Biociências, Universidade Estadual Paulista “Júlio de Mesquita Filho”, Botucatu 18618-689, SP, Brazil; maria.agneis@unesp.br (M.L.G.A.); fabio.seiva@unesp.br (F.R.F.S.); 4 Departamento de Produção Vegetal, Faculdade de Ciências Agronômicas, Universidade Estadual Paulista “Júlio de Mesquita Filho”, Botucatu 18610-034, SP, Brazil; vitor.as.garcia@unesp.br; 5 Departamento de Tecnologia, Centro de Tecnologia, Universidade Estadual de Maringá, Av. Ângelo Moreira da Fonseca 1800, Umuarama 87506-370, PR, Brazil

**Keywords:** ethanol, ultrasonic amplitude, antioxidant potential, chlorogenic acid

## Abstract

The present study aimed to apply ultrasonic probe-assisted extraction (UPAE) using ethanol as a solvent to separate compounds from *Stachys byzantina* leaves. Experimental tests were carried out to investigate the influence of temperature (T) (30, 45 and 60 °C), ultrasonic amplitude (UA) (30, 60 and 90%) and extraction time (ET) (10, 15 and 20 min) on the extraction yield (EY). The extracts obtained at different extraction times were characterized for compound profile, soluble protein content, and antioxidant potential. The cytotoxic effect of the extract was also evaluated. The greatest mass recovery (19.8 wt%) was verified at the highest levels of the variables. The total phenolic compound content and antioxidant potential increased with the application of extraction times of 5 to 20 min, at 60 °C and UA of 90%. The extracts contained ~25 wt% of soluble protein. The extracts showed a predominance of chlorogenic, protocatechuic, and syringic acids. Nicotinic acid was also detected in the extracts, with levels ranging from 11.91 to 13.86 mg/100 g. The fatty acid profile indicated the presence of lauric, palmitic and linolenic acids in higher concentrations, with quantification of squalene, α-tocopherol and β-sitosterol. The ethanolic extract of *Stachys byzantina* showed no cytotoxic effect on HaCaT cells at concentrations up to 200 µg/mL, maintaining cell viability above 70% after 48 h of exposure.

## 1. Introduction

*Stachys byzantina* K. Koch (garden fish) is a perennial herbaceous plant typical of temperate climate regions [1], classified as an unconventional food plant (UFP), which is grown on a small scale and used without prior processing. The phytochemical composition of its leaves is characterized by the presence of secondary metabolites, which give the extract obtained from its leaves antibacterial [2], antioxidants [3] and anti-inflammatory [4] properties.

To commercially enhance the value of UFPs and increase the portfolio of products derived from these matrices, several strategies can be adopted. Among these, the separation of compounds from leaves stands out, especially when coupled with the use of so-called eco-friendly extraction methods. One of the characteristics of these methods is the non-use of non-petroleum-derived solvents [5] and therefore, several requirements are indicated for choosing the solvent considering principles of Green Chemistry [6] and how ethanol fits into them. Furthermore, ethanol is easily removed after extraction due to its low boiling point (78.5 °C) and exhibits extractive capacity for a wide range of compounds present in the plant matrix, such as phenolic acids, flavonoids and proteins [7].

Ultrasonic probe-assisted extraction (UPAE) is an effective method for recovering compounds of interest from diverse plant matrices, which stands out for its superiority over conventional methods in terms of extraction efficiency, processing time, lower solvent consumption and the quality of the extract obtained [8]. Its mechanism is based on the application of high-intensity ultrasonic waves on the plant matrix, promoting acoustic cavitation and consequent formation of transient microbubbles, which intensify the extraction process [9]. The subsequent collapse of these microbubbles generates high magnitude shear forces, promoting the rupture of the cellular structure and facilitating the release and solubilization of the metabolites of interest, without compromising their structural integrity or chemical stability [10]. The use of UAPE presents high efficiency in the extraction of phenols, as evidenced in recent studies [11,12,13,14,15,16,17], which applied ultrasonic power in the range of 50 to 1150 W.

The use of UPAE in the extraction of compounds from *Stachys byzantina* leaves has not yet been explored, with the application of ultrasonic irradiation being limited to the use of an ultrasound bath with indirect contact [4,18,19]. In these studies, ultrasonic power of 320 W to 550 W was applied in the extraction stage, with the focus on extract characterization rather than the effect of operational variables on extract production. Furthermore, the evaluation of extraction in terms of mass yield, a parameter that is crucial for subsequent economic evaluation of the process, was not explored. It is worth highlighting that this is the first study to explore the effect of different conditions on extraction yield using this UFP with UPAE and ethanol as solvent. Therefore, the originality of this study is related to the extraction method applied in conjunction with the selected solvent.

The present study aimed to use UPAE and ethanol as solvent to separate compounds from the leaves of *Stachys byzantina*. Thus, the impact of process variables (temperature, ultrasonic amplitude and extraction time) was investigated to establish the conditions that maximize the extraction yield (EY). The influence of extraction time on the composition of the obtained extract was also evaluated. Finally, the cytotoxicity of the obtained extract was evaluated against HaCaT cells (human keratinocytes).

## 2. Materials and Methods

### 2.1. Materials

The leaves of the *Stachys byzantina* (10 cm ± 0.2) were collected in the morning at June 2024 and voucher specimen were deposited at the Herbarium of the State University of Maringá (registration 41060), Paraná, Brazil. Ethanol (Dinâmica, Indaiatuba, SP, Brazil, purity 95%) was used as a solvent.

The total phenolic content was determined using sodium carbonate (Anidrol, Diadema, SP, Brazil), Folin–Ciocalteu (Dinâmica), methanol (Panreac, Barcelona, Spain), n-hexane (Neon, Rio de Janeiro, RJ, Brazil) and gallic acid (Sigma-Aldrich, St. Louis, MO, USA). The antioxidant potential was determined using: ultrapure water (TE-4007-20, Tecnal, Niort, France), potassium persulfate (Dinâmica, purity ≥ 99.0%), 2,2-diphenyl-1-picrylhydrazyl (DPPH•) (Sigma-Aldrich, purity ≥ 95.0%), 6-hydroxy-2,5,7,8-tetramethylchromane-2-carboxylic acid (Trolox) (Sigma-Aldrich, purity ≥ 97.0%), 2,2′-azino-bis (3-ethylbenzothiazoline-6-sulfonic acid) (ABTS•+) (Sigma-Aldrich, purity ≥ 98.0%). To determine the soluble protein content, the following were used: sodium hydroxide (Anidrol, purity ≥ 97.0%), sodium citrate (Synch, New York, NY, USA, ≥ 99.0%), sodium carbonate (Êxodo Cientifica, Sumaré, SP, Brazil, ≥ 98.5%), copper sulfate (Anidrol, purity ≥ 98.0%). To determine the profile of phenolic compounds, methanol (Merck, Darmstadt, HE, Germany) and formic acid (HPLC grade, Sigma-Aldrich) were used.

For the cytotoxicity assessment, Dulbecco’s Modified Eagle Medium (DMEM, Gibco, Paisley, United Kingdom), fetal bovine serum (Gibco, São Paulo, Brazil), streptomycin (Gibco, São Paulo, Brazil), penicillin (Nova Biotecnologia, São Paulo, Brazil), and 3-(4,5-dimethylthiazol-2-yl)-2,5-diphenyltetrazolium bromide (MTT, Sigma-Aldrich) were used.

### 2.2. Preparation of Raw Material

The leaves were subjected to a sanitation process in running water, followed by removal of residual moisture. Subsequently, these leaves were dried in an oven with air circulation (7lab, SSDic—40) at 40 °C, for 12 h, presenting a moisture content of 7.51 ± 0.01 wt%. The dried leaves were ground (Philips Walita, RI7300, Minas Gerais, Brazil), resulting in a material with a spongy texture, which was stored protected from exposure to light and kept refrigerated.

### 2.3. Ultrasonic Probe Assisted-Extraction (UPAE)

**Figure 1:**
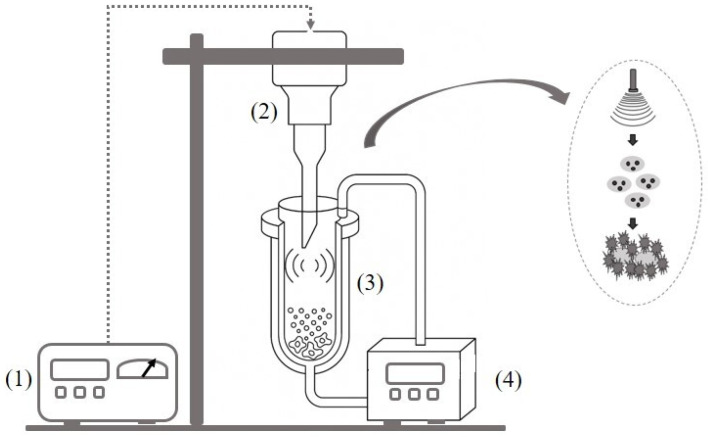
Schematic diagram of the ultrasonic probe-assisted extraction apparatus. (1) Ultrasonic disruptor, (2) Probe-type sonicator, (3) Extraction cell and (4) Heating bath.

Figure 1 shows the experimental apparatus applied to conduct UPAE. In each extraction, 70 mL of ethanol were stored in a jacketed glass cell (100 mL), connected to a thermostatic bath (Polyscience, MX07R-20A11B, Niles, IL, USA) with water recirculation to heat the solvent, whose temperature was monitored by a chemical thermometer (Incoterm, 5021, Rio Grande do Sul, Brazil).

Once the test temperature was reached, 0.7 g of the sample was added to the cell, establishing a sample to solvent ratio of 1:100 (g/mL). The sample to solvent ratio was established to ensure that the entire sample mass is in contact with the solvent. A high-intensity ultrasonic processor (Disruptor QR850, Ultronique, São Paulo, Brazil) with power of 850 W and ultrasonic frequency of 20 kHz was used in the experiments, which was equipped with ultrasonic probe (641 W/cm^2^, 13 mm diameter titanium macro tip). After the extraction period, the solid phase was separated by vacuum filtration (−200 mmHg back pressure), using 0.2 mm thick filter paper (Unifil, Paraná, Brazil). The solvent in the extract was removed, until reaching a constant weight, by rotary evaporation (Fisatom, São Paulo, Brazil) at 50 °C, with a rotation of 20 rpm and a back pressure of −500 mmHg. The extraction yield (EY) was determined from the ratio between the mass of the dry extract obtained and the initial mass of the sample inserted into the extractor (on a dry basis).

An experimental design was adopted, in which the temperature (T) was tested at 30, 45 and 60 °C, the ultrasonic amplitude (UA) was adjusted to 30, 60 and 90% and the extraction times (ET) of 10, 15 and 20 min were considered. In establishing the variable levels, it was considered that higher temperature and time values could cause possible degradation of thermolabile compounds, as well as evaluating the manipulation of the maximum power of the available equipment.

To evaluate the effects of the independent variables (T, UA and ET) on the response variable (EY), analysis of variance (ANOVA) was applied using Statistica^®^ 8.0 software (StatSoft, Inc., Tulsa, OK, USA) considering a 95% confidence interval. Additional experiments were conducted to evaluate the effect of time (5 to 20 min) on the temperature and ultrasonic amplitude conditions that provided greater EY.

The mass yield was obtained using a Soxhlet extractor for comparative purposes. For this, exhaustive extraction was conducted at the boiling point of ethanol for 6 h using a sample-to-solvent ratio of 1:300 (g/mL).

### 2.4. Extract Characterization

For characterization, extracts obtained at different extraction times (5 to 20 min) at 60 °C and ultrasonic amplitude of 90% were selected. Analyses were performed in at least triplicate (n = 3), and mean values were reported followed by standard deviation. Means, when applicable, were compared using ANOVA and Tukey’s test (Statistica^®^ 8.0 software) with a 95% significance level. Prior to each analysis, the samples were diluted in ethanol and heated to 50 °C until completely dissolved.

The colorimetric method involving the Folin–Ciocalteu reagent [20] was used to quantify the content of total phenolic compounds (TPC), from the calibration curve obtained with gallic acid in the concentration range of 0.006 to 0.30 mM (R^2^ > 0.99). The antioxidant potential of the extracts was determined by the DPPH• [21] and ABTS•^+^ [22] methods. A UV-VIS spectrophotometer (Shimadzu, UV 1900, Tokyo, Japan) was used to read the absorbance of the solutions obtained by each method. To quantify the antioxidant capacity, a calibration curve obtained from Trolox at concentrations of 0.05 to 0.95 mM and 0.10 to 2.0 mM was used for the DPPH• and ABTS•^+^ methods, respectively. The soluble protein contents were determined according to Lowry et al. [23] after protein extraction.

For identification and quantification of phenolics, ultra-high performance liquid chromatograph coupled to a triple-quadrupole mass spectrometer (UHPLC-MS/MS, Waters ZTQD Acquity LCMS) was used for chromatographic analysis, consisting of an Acquity H-CLASS UPLC coupled to a Xevo TQD triple-quadrupole mass spectrometer with Z spray™ ESI interface, operating in positive and negative ionization modes (Waters, Milford, MA, USA). Compounds were separated on a Waters Acquity UPLC^®^ BEH C18 column (50 mm × 2.1 mm inner diameter, 1.7 µm particle size). The column was maintained at a temperature of 30 °C, with a total analysis time of 16 min, a flow rate of 0.150 mL/min, and an injection volume of 1.5 µL. Mobile phase A consisted of water with 0.1% formic acid, while mobile phase B was composed of methanol. The elution gradient started with 10% B, increasing to 60% in 3 min. The B percentage was then increased to 80% in 1 min and held for another min. Subsequently, the gradient was increased linearly to 100% B in 5.5 min and held for 1.5 min. Finally, the gradient returned to 10% B in 4.5 min and remained at that value for another 4.5 min to equilibrate the column. Data acquisition was performed using MassLynx™ software (v.4.10) and quantification of the compounds was performed from standard curves obtained from the analytical standards of the compounds in the concentration range of 0.05 to 20 mg/L (R^2^ > 0.99).

The fatty acid profile was determined as reported by Mello et al. [24] with sample injection, after methylation [25], into a Zebron™ ZB-Wax capillary column (Phenomenex, 30 m × 0.25 mm × 0.25 μm, Torrance, CA, USA). The method described by Rosa et al. [26] was used to quantify the compound content by gas chromatography coupled with a flame ionization detector (FID). Both analyses were performed on a gas chromatograph (Shimadzu, GC-2010 Plus) equipped with an automatic injector, and details of the heating ramp and equipment settings can be found in the cited references.

To determine the cytotoxicity of the extract, HaCaT cells (human keratinocytes) were cultured in DMEM with 10% fetal bovine serum, penicillin and streptomycin, and incubated at 37 °C with 5% CO_2_ until reaching 80% confluence. After 24 h of adhesion in 96-well plates, they were treated for 24 and 48 h using different concentrations of the extract (50, 100 and 200 µg/mL). Cell viability was assessed by the MTT assay, with reading at 570 nm (EON Biotek, Winooski, VT, USA) [27].

## 3. Results

### 3.1. Extraction Yield (EY): Effect of Process Variables and Establishment of Maximum Condition

The EY results obtained from the adopted experimental design are shown in Table 1. Based on the results of this table, the analysis of variance (Table S1, Supplementary Material) was generated to evaluate the effects of each variable, as well as their interactions on the response variables.

The fit to the experimental data, considering only the significant terms (*p* < 0.05), is presented in Equation (1).

EY (wt%) = 13.72 + 5.32 T + 1.11 UA + 3.01 ET − 1.53 T × UA + 1.78 T × ET + 1.57 UA × ET + 1.35 T × UA × ET(1)

where T, UA and ET refers to temperature, ultrasonic amplitude and extraction time, respectively.

This equation is considered valid to represent the experimental data, considering that the lack of fit term was not significant (Table S1) and the value of Fcalculated (67.51) > Ftabulated (8.89). The agreement between the experimental and predicted data (R^2^ > 0.99) is also verified, as illustrated in Figure S1 (Supplementary Material). From Equation (1), the maximum EY (19.91 wt%) condition was established: T = 60 °C, UA = 90% (765 W) and ET = 20 min. The verification tests conducted under this experimental condition resulted in an EY of 19.85 ± 0.08 wt%, a value that does not differ from that predicted by the equation (*p* < 0.05).

**Figure 2:**
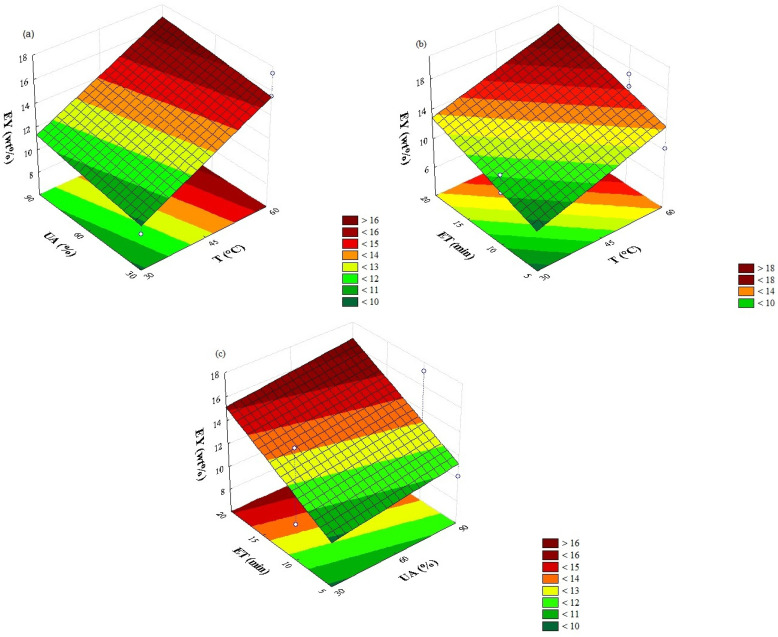
Response surface analysis for the extraction yield (EY) of the ultrasonic probe-assisted extraction of compounds from *Stachys Byzantina* leaves (T: temperature; UA: ultrasonic amplitude; ET: extraction time). Correlative effects of (**a**) UA and T; (**b**) ET and T; and (**c**) ET and UA. Each graph represents the function of the two variables, keeping the third at the center point.

Figure 2 presents the graphical representation, through three-dimensional response surfaces, of the effects observed for the independent variables. From the presented data, it is observed that the linear terms of all variables exerted a positive effect on EY. Figure 2a shows that a UA of 90% was ideal for maximum EY recovery as a function of T of 60 °C. Figure 2b presents an increase in EY proportional to the progressive increase in T in both ET, indicating mass yield when associated with a longer heating duration (ET 20 min; 60 °C), compared to the other temperatures. A similar behavior was observed in Figure 2c, which highlights the positive correlation between UA and ET (90%; 20 min).

#### 3.1.1. Temperature Effect

EY values of 19.85 wt% and 12.99 wt% were obtained using the maximum (60 °C) and minimum (30 °C) temperatures, respectively. The increased extract recovery with increasing temperature is due to the increased solubility and diffusion coefficient of the solvent-soluble leaf constituents. The increase in temperature contributes to a decrease in the viscosity and surface tension of the solvent [8,10].

It should be noted that the intensification of cavitation microexplosions occurs with higher temperature values, increasing the diffusivity and mass transfer of the target compounds from the matrix to the solvent [28] effect evidenced by the significant term of the interaction between T and UA (Equation (1)) and Figure 2a. The use of temperatures in the range of 30 to 60 [11,29,30] was reported for UPAE, with indication of better results at the highest value of this variable, without reduction in the content of phenolic compounds with the increase in temperature.

#### 3.1.2. Effect of Ultrasonic Amplitude

The application of ultrasonic irradiation generated by the macro tip resulted in an increase in the mass of extract obtained (EY), with increments of 14 and 28% (runs 6 and 8; 5 and 7), respectively, considering the variation of UA 30% and 90%. The result obtained can be attributed to the improved mechanical cavitation effect as the power increases, since such an effect can damage the cell wall and matrix tissues, facilitating the penetration of solvents and accelerating the mass transfer rate of the compound from the leaf to the extraction medium [31]. Bhangu, Ashokkumar and Lee [32] reported that frequencies of 20 kHz (as used in this study) can produce huge cavitation bubbles in solvents used for extraction, which collapse rapidly and produce microjets and strong shear forces, facilitating solvent penetration, cell degradation, and high extraction rates. This frequency was indicated as the one that provided the highest recovery in mass, phenolics and flavonoids from bark [13] and leaves [14] from *Commiphora gileadensis*.

González-Silva et al. [33] reported the use of a high-intensity ultrasonic processor with a power of 400 W and obtained a higher content of soluble phenols with an increase in ultrasonic amplitude from 60 to 100%. Mahindrakar and Rathod [34] indicated 134 W as the ideal power for extracting phenolics from waste Syzygium cumini leaves, after evaluating powers of 52, 76, 95, 134 and 202 W.

#### 3.1.3. Effect of Extraction Time

The increase in the time from 10 to 20 min favored higher EY values, with the finding of greater percentage increases in the highest levels of the T and UA variables. Several authors explain that extraction occurs in two distinct stages, characterized by surface washing, in which soluble compounds present on the surface of the plant matrix are readily removed, followed by the diffusion stage, characterized by the migration of active constituents located inside the matrix to the solvent medium. The highest mass recovery rate (from 62 to 90%) occurred in the initial 10 min, indicating that the washing stage was predominant.

The application of high-amplitude acoustic waves over time promotes the degradation of plant cell membranes through physical interactions induced by the formation of gas microbubbles, generating intense mechanical disturbances and favoring the mass transfer of compounds integrated into the plant matrix [35]. In addition, breaks in cell conformation facilitate solvent absorption, resulting in increased compound solubility and intensified mass transfer during the extraction process [8,13], which is due to the driving force of ultrasonic waves, which cause microbubbles to collapse over a longer period of time [36].

The variation in ET from 10 to 20 min resulted in percentage increases of ~62% (runs 4–8) and ~12% (runs 2–6) at 60 °C for UA of 90 and 30%, respectively. This shows that the interaction between the variables contributed to greater process efficiency, as shown in Equation (1). Thus, reducing AU and T in order to minimize energy consumption leads to a reduction in the mass recovery of compounds from the leaves, suggesting that longer extraction times need to be adopted.

### 3.2. Effect of Time on Extract Composition

Table 2 presents the data relating to the characterization of the extracts obtained under the maximum conditions indicated by Equation (1), for temperature and ultrasonic amplitude, at extraction times of 5, 10, 15 and 20 min. The extract obtained after 20 min of extraction showed a higher EY, highlighting the importance of this variable in the mass recovery process. This behavior can be attributed to the greater interaction between the solvent and the plant matrix, promoting more efficient diffusion of solutes, favored by the effects of ultrasound-induced cavitation [10]. Exhaustive extraction using the Soxhlet method indicated an EY of 20.33 ± 0.59 wt%, a value close to that obtained in 20 min at UPAE, indicating that longer extraction times are not required.

The extract obtained after 20 min of extraction showed higher levels of total phenolic compounds, with a higher content of ~84% compared to that obtained after 5 min. This result can be attributed to the longer duration of the sonic cavitation process, favoring the penetration of the solvent into the plant matrix, increasing the release of bioactive constituents [13]. From these results, it can be seen that no degradation of the compounds is observed with the time of exposure to ultrasonic irradiation at a temperature of 60 °C, proving that the interaction between these variables (Equation (1)) is beneficial in the extraction of compounds from the leaves of *Stachys byzantina*.

The TPC content of the extract obtained in this study, at all extraction times evaluated, was higher than previously reported for extracts obtained from *Stachys byzantina* leaves, with levels 0.811 mg GAE/g [19] and 48.24 mg GAE/g [37], which highlights the relevance of the strategy adopted in relation to the choice of technique and solvent. In these studies, the following conditions were used: eutectic solvent composed of L-Proline and levulinic acid (leaf:solvent ratio of 1:16) at 50 °C for 6 min [19] and boiling water, leaf:water ratio of 1:10, for 15 min [37]. Additionally, the extract obtained in this study had a higher TPC content (mg GAE/g) than that obtained from the leaves of other UFPs, such as *Xanthosoma sagittifolium* with 22.34 [38], *Pereskia aculeata* with 40.27 [39] and 66.0 [40], *Moringa oleifera* Lam with 88.66 [41], and *Victoria Amazonica* with 0.52 [42].

From Table 2 it can be seen that the soluble protein content of the dry extract remained around 25 wt%, regardless of the extraction time applied. It is known that the intensity of power and amplitude are directly related, especially in the context of sound waves, and the literature indicates that, generally, the ultrasonication time depends on the intensity of power and the frequency generated in the ultrasonic system [43]. Ampofo and Ngadi [44] reported that short-term high-intensity or high-power ultrasonication is the most advantageous method for obtaining proteins, due to its limited effect on denaturation, energy expenditure, and loss of functional properties. However, in this study, increasing the time did not alter protein levels, indicating that this result may be attributed to the efficiency of UPAE in protein recovery, as evidenced by Mijalković et al. [45], who report that after 5 min of extraction, increasing the time (up to 20 min) did not influence the protein yield obtained from pumpkin leaves.

The antioxidant potential of the extracts followed the same trend observed for TPC content, noting a percentage increase of 28% and 40% for ABTS•+ and DPPH•, respectively, indicating a possible correlation between these properties. Phenols act as hydrogen and electron donors, as well as reducing agents capable of neutralizing reactive oxygen species [46], explaining their synergy with data related to antioxidant analysis. However, the performance of the ABTS•+ and DPPH• assays depends on the balance between polar and nonpolar compounds, the predominant mechanism of radical neutralization, and the solubility of the antioxidant molecules in the extract [47]. This suggests that the percentage increase provided by the DPPH• > ABTS•+ method is due to the predominance of lipophilic compounds in the extract, since the DPPH• radical preferentially captures these constituents, while ABTS•+ has the potential to detect both hydrophilic and lipophilic compounds, explaining the superiority of their levels.

Table 3 shows that nine compounds were identified in the extracts, in which quercetin was the compound identified with the lowest concentration. In general, the compound levels in the extract were influenced by the longer contact time of the leaves with the solvent. The combined analysis of EY, TPC content, antioxidant potential, and compound profile data demonstrates that increasing the time favors not only greater mass recovery of the leaves, but also the quality of this extract in active compounds. Although the individual content of some compounds decreased with extraction time, such as caffeic and protocatechuic acids, the total content of compounds increased. Therefore, considering EY (Table 2) and total compounds (Table 3), it is possible to obtain an increase in compound recovery of approximately 2.3 times with an increase in time from 5 to 20 min.

Chlorogenic, syringic and protocatechuic caffeic were the major phenolic acids in the extracts, representing ~78.82, 7.36, and 7.12% of the total extract composition (ET of 20 min), respectively. Benedec et al. [17] indicated a higher content of chlorogenic acid (~95.16%) in the total composition of extracts from *Stachys Byzantina* leaves and Bahadori et al. [3] reported extracts with 578 mg/100 g for chlorogenic acid. This phenolic acid presented a higher concentration in the extracts obtained at 15 and 20 min, giving them greater antioxidant potential, as shown in Table 2. A strong correlation between chlorogenic acid content and ABTS•+ (0.895) and DPPH• (0.936) values was reported by Young et al. [48] in extracts of *Dendropanax morbifera* leaves.

Nicotinic acid (vitamin B3 complex) was identified in the extracts, which stands out for its lipid-lowering action, raising HDL cholesterol levels and reducing triglycerides, as well as LDL cholesterol [49]. The presence of nicotinic acid in the leaves and extracts of *Stachys byzantina* has not yet been reported. Passos et al. [39] identified nicotinic acid (8.3/100 g dry extract) as one of the main compounds present in the extracts of the *Pereskia aculeata* leaf.

### 3.3. Identification of Compounds Present in the Extract by Gas Chromatography

The extract, obtained in 20 min of extraction, 60 °C, and applying 90% of UA, showed a predominance of lauric, palmitic and linolenic acids, which represented ~70% of the fatty acid composition, as shown in Table 4.

The fatty acid composition suggests a mixed profile for the extract, with the participation of an unsaturated fatty acid (linolenic), with biological activity and antioxidant functionality, which, combined with what was previously demonstrated and discussed (Table 2 and Table 3), reiterates its bioactivity, as well as the presence of saturated fatty acids (lauric and palmitic), which generally contribute to greater thermal and oxidative stability [50]. In general, the fatty acid profile of the analyzed extract showed a predominance of saturated fatty acids, which totaled approximately 58.5% of the total fraction. The ratio of ~0.5 between linoleic and linolenic acid (omega 6/omega 3) indicates a balanced nutritional profile, an alternative for nutraceutical and functional applications that favor anti-inflammatory properties. However, the presence of trans fat (trans-vaccenic acid) may require caution in certain applications.

The extract presented α-tocopherol and β-sitosterol in its composition, which was previously reported by Lima et al. [19] and Aminfar et al. [51], respectively, in extracts obtained from *Stachys byzantina* leaves. Squalene was identified and quantified at a low concentration and has not been previously reported.

### 3.4. Cytotoxicity Analysis

In the cytotoxicity analysis of the extract (189.69 mg GAE/g), evaluated at different concentrations (50 to 200 µg/mL), it was observed that, regardless of the concentration, there was no cytotoxic effect in the first 24 or 48 h of exposure to the extract. At 50 µg/mL, the cell viability values were 106.73 ± 9.82% and 103.99 ± 22.23%, for 24 and 48 h, respectively. At the concentration of 100 µg/mL, the values were 105.28 ± 8.56% (24 h) and 81.92 ± 2.86% (48 h). At the highest concentration evaluated (200 µg/mL), cell viability was 100.96 ± 5.32% after 24 h and 71.89 ± 3.92% after 48 h. These results indicate that the extract did not induce significant cytotoxicity in HaCaT cells under the conditions tested, with cell viability values above 100% at 24 h and remaining above 70% even after 48 h of exposure. Although the viability at 48 h and 200 µg/mL (71.89 ± 3.92%) suggests a possible mild sub-cytotoxic effect, the overall response indicates good cellular tolerance to the extract [52].

*Stachys byzantina* leaves are traditionally used in Brazilian cuisine, especially after frying, and to date, there are no reports in the literature of adverse effects related to their consumption. The results obtained can be compared to previous work related to the viability of HaCaT cells when exposed to the extract of *Stachys byzantina* leaves, such as Lima et al. [4] who report ~90% cell viability after 24 h of exposure to the extract at a concentration of 12.5 µg/mL (34.30 mg tannic acid equivalents/g). Additionally, the cell viability values were higher than those reported by Leite et al. [53] who indicated values of 51.6% after 48 h of exposure with application of extract (116.30 mg tannic acid equivalents/g) at 100 µg/mL. Bahadori et al. [3] evaluated the cytotoxic effect of essential oil from different Stachys species (hedgenettle or woundwort) and reported that the extract of *Stachys byzantina* did not show any considerable cytotoxic activity in cancer cells (IC50 > 1000 mg/mL). Yousefbeyk et al. [37] also reported that particles obtained by green synthesis of *Stachys byzantina* (19.14 mg GAE/g) do not present toxic effects in the MTT assay.

As evidenced in Table 2, Table 3 and Table 4, the extract of *Stachys byzantina* leaves contains several compounds which may be related to the observed cytotoxic effects. Ha et al. [54] indicated that chlorogenic and caffeic acids at concentrations of 35.43 and 18.01 µg/mL, respectively, did not show any effect on cell viability, as determined by the MTT assay, after 48 h of exposure. These findings suggest that possibly the effect observed for the extract of *Stachys byzantina* leaves, at 48 h of exposure, does not correspond to the concentration of this compound, which were applied at the maximum concentrations of 1.00 and 0.023 µg/mL, respectively.

## 4. Conclusions

This study demonstrated the effectiveness of UPAE combined with a green solvent (ethanol) for recovering bioactive compounds from *Stachys byzantina* leaves. The mass recovery of the extract ranged from 9.12 to 19.85 wt%, with the highest yield obtained at 60 °C, 20 min, and 765 W. Except for soluble protein content, increasing the extraction time enhanced the extraction yield, resulting in higher TPC and, consequently, greater antioxidant potential. Prolonged extraction also improved the levels and quality of the profiled active compounds. The fatty acid and minor compound composition supported the classification of the extract as functional, antioxidant, lipidic, and thermostable. The cytotoxicity assay showed that the extract did not induce significant cytotoxic effects in HaCaT cells, maintaining viability above 70% even at the highest concentration tested. While this result indicates good cellular tolerance under the evaluated conditions, it represents an initial step in the toxicological assessment of *S. byzantina* extracts. Therefore, this work should be considered a first stage in the experimental evaluation of this species. Future studies, including in vitro assays on additional cell types and in vivo investigations in experimental models, will be essential to confirm both the biological efficacy and toxicological safety of the extract. Such studies will provide a stronger scientific basis for potential cosmetic, nutraceutical, and pharmaceutical applications of *S. byzantina* extracts.

## Figures and Tables

**Table 1:** Experimental conditions and results obtained from the extraction yield (EY).

Run	T (°C)	UA (%)	ET (min)	EY (wt%)
1	−1 (30)	−1 (30)	−1 (10)	9.12
2	1 (60)	−1 (30)	−1 (10)	15.54
3	−1 (30)	1 (90)	−1 (10)	11.54
4	1 (60)	1 (90)	−1 (10)	12.21
5	−1 (30)	−1 (30)	1 (20)	10.13
6	1 (60)	−1 (30)	1 (20)	17.41
7	−1 (30)	1 (90)	1 (20)	12.99
8	1 (60)	1 (90)	1 (20)	19.85
9 (C)	0 (45)	0 (60)	0 (15)	13.61
10 (C)	0 (45)	0 (60)	0 (15)	14.21
11 (C)	0 (45)	0 (60)	0 (15)	14.23

T: temperature; UA: ultrasonic amplitude; ET: extraction time.

**Table 2:** Effect of extraction time on extraction yield (EY) and composition of phytochemical extract at 60 °C and applying 90% of ultrasonic amplitude.

Property		Extraction Time (min)			
		5	10	15	20
EY (wt%)		10.19 ^a^ ± 0.21	12.21 ^b^ ± 0.51	14.63 ^c^ ± 0.28	19.85 ^d^ ± 0.08
TPC content (mg GAE/g)		101.66 ^a^ ± 0.28	130.11 ^b^ ± 0.28	153.48 ^c^ ± 0.29	189.69 ^d^ ± 0.27
Antioxidant potential (µmol TEAC/g)	ABTS•^+^	549.33 ^a^ ± 4.71	605.90 ^b^ ± 4.64	682.67 ^c^ ± 4.71	707.94 ^d^ ± 7.65
	DPPH•	414.23 ^a^ ± 0.01	455.08 ^b^ ± 2.32	531.00 ^c^ ± 4.71	581.01 ^d^ ± 4.01
Soluble protein content (g/100 g)		24.13 ^a^ ± 0.82	25.02 ^a^ ± 0.33	24.98 ^a^ ± 0.55	25.47 ^a^ ± 0.47

GAE: gallic acid equivalent. TPC: total phenolic compounds. TEAC: Trolox Equivalent Antioxidant Capacity. Averages followed by the same letters (in each line) do not differ statistically (*p* < 0.05).

**Table 3:** Compounds identified by UHPLC-MS/MS in the phytochemical extract obtained at 60 °C and applying 90% of ultrasonic amplitude.

Compound (mg/100 g)	Extraction Time (min)			
	5	10	15	20
Gallic acid	5.64 ^a^ ± >0.01	6.26 ^b^ ± 0.04	5.65 ^a^ ± 4.68	5.76 ^a^ ± 0.01
Caffeic acid	16.55 ^a^ ± 0.44	24.20 ^b^ ± 0.25	17.51 ^c^ ± 0.04	11.64 ^d^ ± 0.39
Chlorogenic acid	411.76 ^a^ ± 4.95	407.07 ^a^ ± 9.56	484.72 ^b^ ± 0.05	502.88 ^c^ ± 8.26
Protocatechuic acid	42.93 ^a^ ± 0.83	56.43 ^b^ ± 1.80	48.98 ^c^ ± 0.51	45.44 ^d^ ± 0.29
*p*-coumaric acid	2.51 ^a^ ± 0.01	3.00 ^b^ ± 0.02	2.72 ^c^ ± 0.61	2.35 ^d^ ± >0.01
Ferulic acid	6.48 ^a^ ± 0.31	6.88 ^b^ ± 0.11	7.70 ^c^ ± 0.58	8.14 ^d^ ± 0.61
Syringic acid	37.70 ^a^ ± 0.06	42.37 ^b^ ± 0.15	38.15 ^a^ ± 1.33	46.94 ^d^ ± 1.33
Nicotinic acid	11.91 ^a^ ± 0.19	14.36 ^b^ ± 0.24	14.03 ^b^ ± 0.75	13.86 ^b^ ± 0.58
Quercetin	1.08 ^a^ ± 0.01	1.12 ^b^ ± 0.05	1.14 ^b^ ± >0.01	1.09 ^a^ ± 0.05
Total	536.59 ^a^ ± 0.21	561.71 ^b^ ± 12.24	620.60 ^c^ ± 8.55	638.10 ^d^ ± 11.52

Averages followed by the same letters (in each line) do not differ statistically (*p* < 0.05).

**Table 4:** Fatty acid profile and compounds quantified by GC-FID of the phytochemical extract obtained at 60 °C, 20 min and applying 90% of ultrasonic amplitude.

Fatty acid (%) ^1^	Lauric	23.06 ± 0.11
	Myristic	8.37 ± 0.03
	Palmitic	22.40 ± 0.03
	Stearic	4.67 ± 0.12
	trans-vaccenic	6.58 ± 0.25
	Linoleic	11.13 ± 0.64
	Linolenic	23.80 ± 0.45
Compounds quantified by GC-FID (mg/100 g) ^2^	squalene	19.20 ± 1.48
	α-tocopherol	127.12 ± 15.91
	β-sitosterol	82.10 ± 5.8

^1^ percentage in normative area of the chromatogram obtained by injecting the sample into a Zebron™ ZB-Wax capillary column. ^2^ compounds quantified from the calibration curve obtained from the chromatographic standards.

## Data Availability

The original contributions presented in this study are included in the article/supplementary material. Further inquiries can be directed to the corresponding authors.

## Supplementary Materials

The following supporting information can be downloaded at: https://www.mdpi.com/article/10.3390/plants14233636/s1; Table S1. Analysis of variance of the regression model for extraction yield (EY). Figure S1. Predicted values versus observed values for adequacy of the predictive equation for extraction yield (EY) of the ultrasonic probe-assisted extraction of compounds from *Stachys byzantina* leaves.
