# Supplementary material for: Ethanolic Extract of Stachys byzantina Leaf: Optimization of Ultrasonic Probe-Assisted Extraction and Characterization
Source: Plants (Basel). 2025 Nov 28;14(23):3636. doi: 10.3390/plants14233636 (PMC12694022; doi:10.3390/plants14233636)
Supplement: Supplementary file 1 [file plants-14-03636-s001.zip › plants-3972128-supplementary.pdf]

Supplementary Material

Table S1. Analysis of variance of the regression model for extraction yield (EY).

Factor	Sum of squares	Mean square	F	p^a
T	56.65	56.65	456.43	0.002
UA	2.47	2.47	19.94	0.047
ET	18.09	18.09	145.73	0.007
T * UA	4.67	4.67	37.59	0.026
T * ET	6.32	6.32	50.90	0.019
UA * ET	4.91	4.91	39.59	0.024
T* UA * ET	3.63	3.63	29.25	0.032
Lack of fit	0.37	0.37	2.96	0.227
Pure Error	0.25	0.12		
Total	97.37			

[a] statistical significance ($p < 0.05$). T: temperature; UA: ultrasonic amplitude; ET: extraction time.

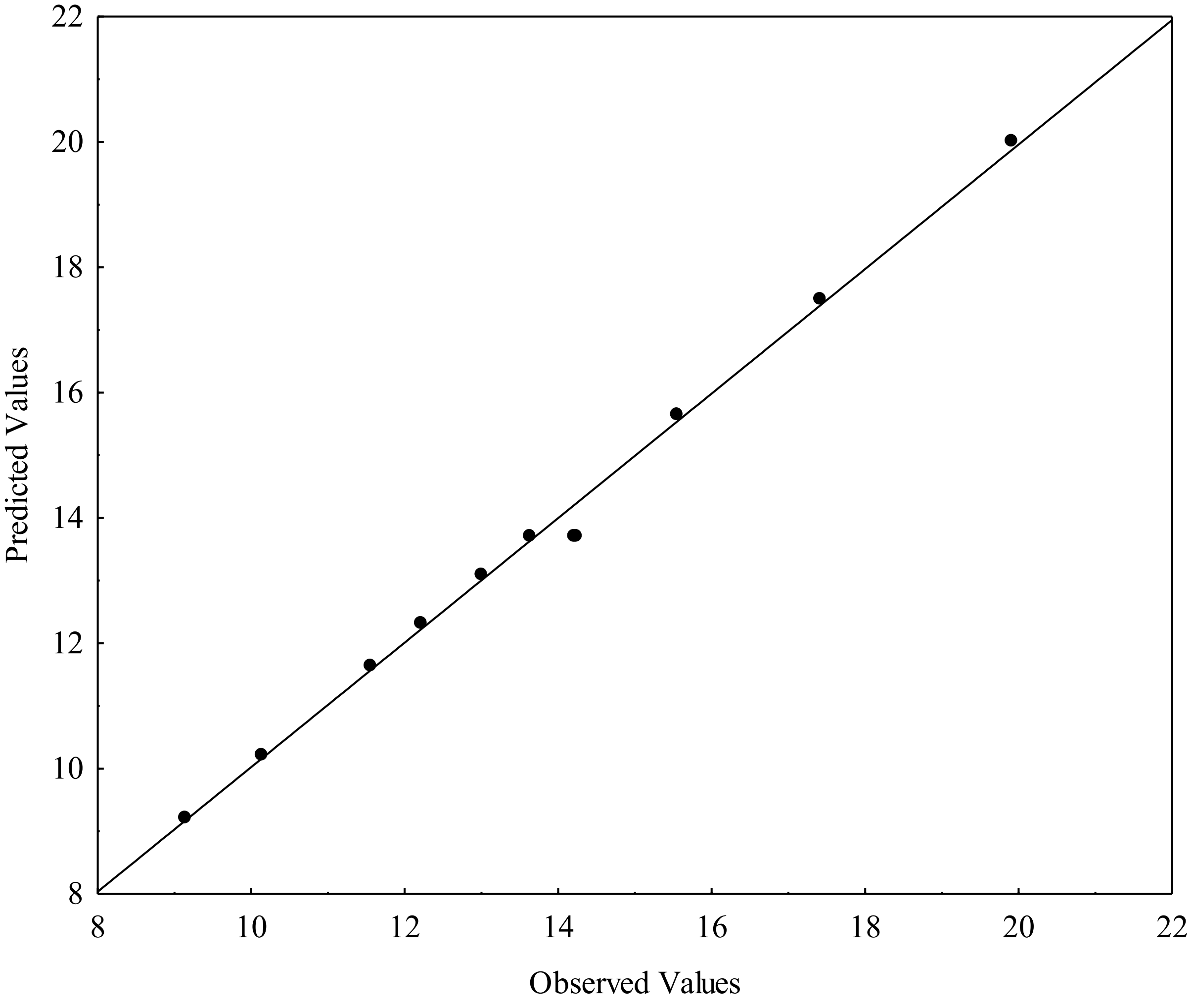

Figure S1. Predicted values versus observed values for adequacy of the predictive equation for extraction yield (EY) of the ultrasonic probe-assisted extraction of compounds from *Stachys Byzantina* leaves.
